# Emerging consensus on the mechanism of polyspecific substrate recognition by the multidrug transporter P-glycoprotein

**DOI:** 10.20517/cdr.2019.22

**Published:** 2019-09-19

**Authors:** Di Xia, Fei Zhou, Lothar Esser

**Affiliations:** Laboratory of Cell Biology, National Cancer Institute, Center for Cancer Research, National Institutes of Health, Bethesda, MD 20892, USA.

**Keywords:** P-glycoprotein, multidrug resistance, substrate polyspecificity, structure, ABC transporter

## Abstract

P-glycoprotein (P-gp or ABCB1) is a member of the broad family of ABC transporters. P-gp participates in the establishment of physiological barriers limiting cellular access of a large number of toxic compounds. It thus plays important roles in the pharmacokinetics of these compounds. Cancer cells and cells infected by viruses exploit the presence of P-gp to fend off drug treatment, rendering them multidrug-resistant. Overcoming multidrug resistance caused by expression of ABC transporters has gained increasing attention in the field of drug development. Recently, studies of P-gp, especially from structural investigations by both cryo-electron microscopy and X-ray crystallography, have provided high-resolution mechanistic details for the function of this transporter. Structures with increasing resolution and accuracy in various substrate- and inhibitor-bound forms are available for analysis and a consensus on the mechanism of substrate polyspecificity is emerging. The use of new structural information may aid development of P-gp inhibitors as well as compounds that may bypass P-gp action.

## Introduction

Multidrug resistance (MDR), the simultaneous development of cellular resistance to multiple anticancer drugs, remains a major obstacle in effective treatment of cancer by chemotherapy^[1]^. MDR is also a known problem in treating microbial infections. Although multiple mechanisms are known in MDR development, one well-studied mechanism is the overexpression of ATP-dependent efflux pumps, represented by ABC transporters such as P-glycoprotein (P-gp or ABCB1). MDR conferred by the ABC family of efflux transporters is achieved by pumping a wide range of drugs out of cells, thus reducing intracellular concentrations required for cytotoxicity^[2]^. The level of P-gp expression has been found to correlate with poor clinical response to chemotherapy in patients^[3]^. Contrary to its role in MDR, P-gp also plays an important physiological role in protecting tissues from potential environmental toxins by participating in various physiological barriers such as the endothelial cells lining the gastrointestinal tract, the blood-brain barrier, and the placenta-blood barrier. An extraordinary property of P-gp is its ability to transport a large number of structurally and chemically unrelated compounds. Understanding how P-gp recognizes and transports these compounds, how it couples the expenditure of ATP hydrolysis to substrate translocation, and how its activity can be modulated requires an accurate and detailed knowledge of the structure of the protein at atomic resolution. Most importantly, it is believed that obtaining correct answers to these questions can help control the activities of P-gp in clinical settings for more effective delivery of drugs into cancer cells by: (1) rational development of novel, targeted therapeutics that bypass P-gp’s action; (2) screening for drugs that reverse the action of P-gp; and (3) looking for compounds that induce cytotoxicity in the presence of P-gp. In this article, we review recent efforts in the field to understand, both biochemically and structurally, the mechanism of function of mammalian P-glycoprotein, focusing on the mechanism of substrate specificity.

## Cellular function of P-glycoprotein

It was soon realized, after the first successful chemotherapy of human cancer, that drug resistance was going to be a major problem in cancer treatment^[2,4,5]^. Combining multiple drugs with different modes of action did not solve this problem, since cancers are capable of developing resistance simultaneously to many different anti-cancer drugs, for which the term MDR was coined. The hope of circumventing MDR in order to improve the efficacy of cancer therapy led to extensive investigations into the mechanisms of MDR and the search for potential agents that are capable of reversing MDR.

As early as 1968, cross resistance to a number of cancer drugs in mouse leukemic cells was reported^[5]^. By 1974, multidrug-resistant Chinese hamster ovary (CHO) cells were isolated and the MDR phenotype was demonstrated, correlating with the reduced drug accumulation inside the cell, thought to be the result of alterations in membrane permeability^[6]^. Resistance to multiple drugs was also observed in Ehrlich ascites tumor cells^[7]^. Further characterization of multidrug-resistant CHO cells showed that the MDR exhibited by CHO cells is energy dependent^[8]^ and MDR was found with correlate with the expression of a surface glycoprotein with an apparent molecular weight of 170 kDa, which was termed the permeability glycoprotein or P-glycoprotein (P-gp)^[9]^. P-gp was subsequently purified from plasma membrane vesicles of resistant CHO cells in 1979, accounting for 3%-4% of total membrane proteins^[10]^. Thus, the MDR phenotype appeared to correlate with the expression of P-gp. Indeed, it was shown that sensitive cells could become resistant by transfecting them with DNA from a resistant line. Furthermore, the expression levels of P-gp in the resistant lines correlated with amplification of the *P-gp* gene^[11]^. However, this is not to say that the control of *P-gp* gene expression is now understood, although several lines of evidence suggest that expression of *P-gp* gene may be regulated^[12]^. For example, at the transcription level, the human MDR1 (*ABCB1*) gene is expressed at a very high level in the adrenal gland, at high levels in the kidney, at intermediate levels in the liver, jejunum, colon and rectum, and at low levels in many other normal organs^[13]^.

The clinical relevance of the expression of P-gp in multidrug-resistant models was investigated by Dano *et al*.^[4]^ in 1972. They found that cross resistance to multiple drugs occurred once a tumor was treated with any one drug. By 1979, a multidrug-resistant human cancer cell line was isolated that showed high expression of a surface glycoprotein that made the cell line prone to aggregation^[14]^. The expression of this surface glycoprotein was shown to be widespread in various cancer cell types^[15]^. The association of the MDR phenotype with the presence of a surface membrane protein (P-gp) and with the expression of the *MDR1* gene product was established, using multidrug resistant human KB carcinoma cells that harbor the *MDR1* gene and also express P-gp, to show that the cDNAs encoding P-glycoprotein cross-hybridize with the cDNAs of multidrug-resistant cells^[16]^. The relevance of P-gp expression in clinical MDR was further strengthened by the detection of P-gp using a specific monoclonal antibody in two clinical samples from patients with ovarian cancer^[17]^. Most importantly, it was shown that the MDR phenotype in cancer cell lines could be reversed by verapamil, raising the expectation that clinical MDR could be circumvented^[18]^. Indeed, in a clinical trial for patients with acute myeloid leukemia, it was shown that patients treated with the MDR inhibitor cyclosporine A plus chemotherapy displayed a significant survival advantage^[19]^.

Fojo *et al*.^[13]^ found that the function of P-gp was not limited to conferring MDR in cancers, as the mRNA of P-gp was also found present in various normal tissue types. In particular, MDR1 gene expression at the protein level could be detected using the specific monoclonal antibody MRK16 in different tissues^[20]^, showing the protein levels that were corroborated with that of mRNA. Furthermore, P-gp was found to be abundantly expressed at physiological barriers such as the placenta-blood^[20]^ and the blood-brain barriers^[21]^. It was thus speculated that P-gp had evolved as a general protection mechanism to keep toxic xenobiotics from entering the body through the intestines, to excrete such xenobiotics via the kidney and liver if they were absorbed, and to protect vulnerable tissues from circulating toxins by the blood-brain, blood-testis/ovary, and placental barriers^[3]^. Indeed, P-gp was discovered to be a major factor in reducing the oral availability of amphipathic drugs such as Taxol and P-gp^[22]^ knockout mice were found to be viable under normal conditions but displayed severe sensitivity to toxic environments^[23]^.

### Biochemical characterizations of P-glycoprotein

Tremendous efforts to characterize P-gp have been made in the hope that the activity of P-gp could be curbed in various cancers. By 1986, human, mouse and Chinese hamster *P-gp* genes had been cloned and their primary sequences obtained^[24-26]^. Two genes relating to the MDR phenotype were identified in human, termed MDR1 and MDR2^[27]^ with MDR1 coding for P-gp. Mice and hamsters have three *MDR* genes with two of them close homologs of human MDR1, termed mdr1a and mdr1b. P-gp was shown to be a member of the ABC transporter family, featuring a long polypeptide chain (1280 amino acid residues for the human P-gp) with two homologous halves, each consisting of a predicted transmembrane domain (TMD) and nucleotide-binding domain (NBD). Efforts to obtain purified P-gp for a variety of *in vitro* studies ensued. An initial attempt to purify human P-gp was conducted using drug-selected cell lines by immunoaffinity chromatography in a single step, resulting in a protein with its ATPases activity preserved^[28]^. Heterologous expression of P-gp in insect cells yielded membrane proteins without extensive glycosylation but with the drug-binding activity intact^[29]^. With partially purified protein available, many biochemical characterization experiments followed. Isolated membrane vesicles from multidrug resistant cells were shown to accumulate drugs in an ATP-dependent manner^[30]^. Of particular interest, partially purified P-gp possesses drug-stimulated ATPase activity when reconstituted into lipid^[31,32]^ and the ATPase activity of P-gp is inhibited by thio-reactive NBD-Cl and N-ethylmaleimide and by vanadate^[33]^.

Subsequent biochemical and functional studies of P-gp centered on more detailed investigations of the ATP hydrolysis cycle and the drug-binding pocket. Like many other mechanistic studies, high quality, isolated P-gp was required for the establishment of a clean, *in vitro* assay system, leading to a further push for more efficient, heterologous recombinant expression systems for either human or mouse P-gp and for better purification procedures^[32,34]^. When highly purified P-gp became available, a flurry of biochemical characterizations followed, including *in vitro* reconstitution into lipid^[35]^, drug-stimulated ATPase activity^[31]^, and vanadate-promoted trapping of ADP^[36]^. The results of these experiments led to the conclusion that both ATP sites of P-gp are important for function; they interact with each other and ATP hydrolysis most likely alternates between the two sites^[37]^.

The search for the molecular basis of P-gp’s ability to recognize and transport a seemingly unlimited number of structurally and chemically diverse compounds has been another major focus of P-gp research. To map the substrate-binding site or to identify which amino acid residues interact with P-gp substrates or inhibitors, multiple techniques were employed. One was to construct a cysteine-less P-gp variant, in which single or multiple cysteine residues were subsequently re-introduced for covalent interaction with thio-reactive agents or substrates to identify drug-binding sites^[38-42]^. Another method used radioactive photo-affinity substrates to cross-link P-gp, leading to the proposal of two or more drug-binding sites in P-gp^[43-45]^. Additionally, it was found that the apparent affinities of P-gp for the anticancer drugs actinomycin D and paclitaxel are orders of magnitude higher in the presence of lipids than they are in a detergent environment^[46]^, suggesting a possible role of lipids in substrate recognition.

Based on extensive experimental analysis, various mechanistic models have been proposed for the function of P-gp. These models can be grouped into the following categories: (1) for substrate entry into the transporter, the Hydrophobic Vacuum Cleaner Model was proposed^[47-49]^, which suggests that drugs entering P-gp is mediated by the membrane bilayer due to differential partitioning of hydrophobic drugs; (2) how substrates interact with the drug-binding site has been the subject of extensive studies. Using a photoactive substrate and an allosteric modulator, the existence of two drug binding sites, N- and C-terminal sites, was demonstrated, leading to the Two-Site Transport Model (ON and OFF)^[42]^. Consistent with this model, labeling by a photoaffinity analog of a drug-substrate showed altered affinity for the drug in the vanadate (Vi)-trapped transition state versus the nucleotide-free state, which became a surrogate assay to monitor the conversion of the high affinity “ON” site to the low affinity “OFF” site^[50]^. The Flippase Model, derived from the resemblance of the drug transport process to lipid flipping, proposes that drugs from the inner leaflet are flipped to the outer leaflet by the action of P-gp, where they can diffuse into the aqueous environment^[47]^. In contrast to the Two-Site and Flippase models, Loo and Clarke found, through the use of thioreactive substrates and P-gp variants with systematically introduced cysteine residues to the TM helices, that for each substrate multiple TM helices participate in binding and the protein undergoes conformational changes. Thus, they proposed the Induced-Fit Model^[41]^; (3) to exit the transporter, it is proposed that drug substrates undergo a rehydration process^[51]^; (4) the transport cycle of P-gp is driven by ATP binding and hydrolysis cycles in the two NBDs. An Alternate-Site Model was proposed to account for the observed asymmetry and stoichiometry in nucleotide binding of the two NBDs^[37]^. Furthermore, the Constant Contact Model proposes a close proximity of the two NBDs during the ATP hydrolysis cycle due to the fact that intracellular ATP is abundant and due to the observation that the two NBDs are tightly associated in the presence of nucleotides, as seen in bacterial ABC transporters^[52-54]^; and (5) the elucidation of the mechanisms dealing with the coupling between ATP hydrolysis and drug translocation requires the establishment of the transport stoichiometry or the ratio between the number of ATP molecules hydrolyzed per drug transported, which has been reported to be 2^[55]^, with one ATP used for drug transport and the second one for resetting the transporter conformation^[56]^. A Half-Coupled Mechanism was proposed to account for the polyspecificity of substrate recognition by and for the uniform substrate-stimulated ATPase rate observed for P-gp^[57]^. Considering the biochemical evidence of multiple sites for drug interactions, van Veen *et al*.^[58]^ proposed a Two Cylinder-Engine Model for the coupling between substrate transport and ATP hydrolysis. ATP binding and hydrolysis were considered as a switch to control substrate binding and release in the ATP Switch Model^[59]^. Clearly, each of these models captures certain aspects of P-gp function, but we still lack a unifying mechanistic model, for which authentic structural information of P-gp is required.

### A brief overview of high-resolution structures of P-gp

Although the general principle governing ligand transport across a biological membrane was proposed a long time ago as the alternate access model^[60]^, the actual implementation of this model for different transporters varies considerably. Despite much progress made in understanding physiological and mechanistic aspects of P-gp function, a number of important questions remain unanswered. Some of the questions with respect to the mechanism of P-gp function are listed below: (1) there is biochemical evidence that P-gp retains nucleotide asymmetrically during its reaction cycle, one per P-gp molecule at a time^[61-63]^, which is consistent with the Alternate-Site Model. However, the question of whether this bound nucleotide prefers NBD1 or NBD2 has not been resolved; (2) what are the structural features of P-gp that enable its substrate polyspecificity and the simultaneous binding of multiple substrates (allocrites)? (3) what is the structural basis for basal ATPase activity, ATP hydrolysis in the absence of substrates, and for the substrate-stimulated ATPase activity? (4) P-gp is known for its structural flexibility, the significance of which remains to be learnt. Specifically, how to relate the various conformations of P-gp to the states observed during its catalytic cycle is a challenge that we face today. To address these mechanistic questions, we must obtain a structural framework that contains atomic details of all the parts needed for P-gp function.

**Figure 1:**
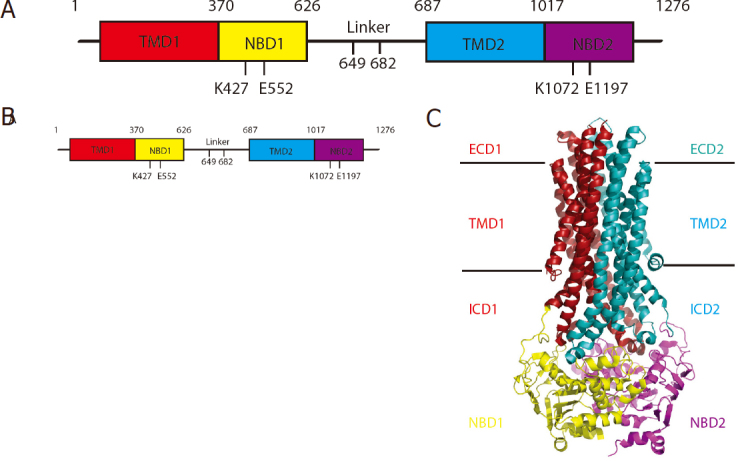
Structures of P-glycoprotein in different conformations (A) Domain organization of P-gp according to mouse residue numbering. K427 and K1072 are lysine residues of the Walker A motifs of NBD1 and NBD2, respectively. E552 and E1197 are glutamate residues of the Walker B motifs of NBD1 and NBD2, respectively. (B) Cartoon rendition of the structure of P-gp in the inward-open conformation based on the deposited mouse P-gp structure (PDB:5KPD). The inward-open conformations are defined by the separation of the NBD1 and NBD2 and the drug-binding cavity in the TMD accessible to the cytosol. The horizontal lines represent the boundary of the membrane bilayer. Color codes for individual domains are indicated. (C) Cartoon representation of P-gp in the outward-open conformation based on the cryo-EM structure of human P-gp bound with ATP (PDB:6C0V). The outward-open conformation is defined by the close contact of the two NBDs with ATP occluded in the nucleotide-binding sites and the drug-binding cavity of TMD accessible from the extracellular space

For human P-gp, the protein is encoded by a single polypeptide chain of 1280 amino acid residues of two similar halves joined by a flexible linker; it consists of four functional domains: TMD1, NBD1, TMD2, and NBD2 [Figure 1A]. High-resolution structures of P-gp available in the protein databank (PDB) were determined either by X-ray crystallography or more recently by cryo-electron microscopy (Cryo-EM) [Table 1]. Despite the larger number of structures determined by the diffraction method, obtaining high quality P-gp crystals has proven to be very challenging, especially for human P-gp (*h*P-gp). Structures of P-gp were first obtained using the diffraction method for mouse P-gp (*m*P-gp)^[64]^, and were later revised^[65]^ in light of the publication of the homologous structure from *C. elegans* P-gp (*Ce*P-gp)^[46]^. These structures share a common feature of having an inward-open conformation [Figure 1B]. A structural model for *h*P-gp in the inward-open conformation has thus been constructed based on the structure of *m*P-gp, which has a sequence identity of 87% to the *h*P-gp. Improvement in resolution of the *m*P-gp structures was further attempted when the linker that connects the two halves of P-gp was shortened by 34 residues^[66]^, because this distinct 70-residue long linker has never been completely observed in any of the structures determined to date and was thought to interfere with conformational uniformity of P-gp. To model the outward-open conformation for *h*P-gp, a number of bacterial ABC transporter structures were used because these structures were determined in the outward-open conformations (Sav1866 and MsbA)^[67,68]^. Since the sequence identities between *h*P-gp and bacterial transporters are low, the quality of these models is poor and considered insufficient to provide necessary guidance for further mechanistic analysis of *h*P-gp in the realm of substrate and modulator interactions. This situation changed when the structure of *h*P-gp in the open-outward conformation determined by cryo-EM was reported in 2018^[69]^. The power of using cryo-EM was again demonstrated for the high-resolution structure determination of a mouse-human chimeric P-gp in complex with the monoclonal antibody UIC2 in the open-inward conformation both in detergent and in nanodisc and the human P-gp bound with an anticancer drug taxol imbedded in nanodisc^[70,71]^.

**Table 1:** Published eukaryotic P-gp structures

PDB	Species	Gene name	Mutation	SG^a^	Conformation	Bound ligand	Res (Å)	Rf^b^	Release Date	Method	Ref
6QEX	H. sapiens	ABCB1	In nanodics		Inward open	MabUIC2/Fab,Taxol	3.6		2019-02-27	Cryo-EM	Alam *et al*., 2019
6QEE	M. musculus	Mdr1a	ECL replaced with ABCB1, E552/1197Q in nanodics		Inward open	MabUIC2/Fab, Zosuquidar	3.9		2019-02-27	Cryo-EM	Alam *et al*., 2019
6FN1	M. musculus	mdr1a	ECL replaced with ABCB1 sequence, S559/1204C		Inward open	MabUIC2/Fab, Zosuquidar	3.58		2018-02-21	Cryo-EM	Alam *et al*., 2018
6FN4	M. musculus	mdr1a	ECL replaced with ABCB1 sequence, S559/1204C		Inward open	MabUIC2/Fab,	4.14		2018-02-21	Cryo-EM	Alam *et al*., 2018
6C0V	H. sapiens	ABCB1	E556/1201Q		Outward open	ATP•Mg to both NBDs	3.4		2018-01-31	Cryo-EM	Kim and Chen, 2018
5KPD	M. musculus	mdr1a	ΔLnk34, E552/1197Q	P2_1_2_1_2_1_	Inward open	None	3.35	0.283	2016-12-14	X-ray	Esser *et al*., 2017
5KPJ	M. musculus	mdr1a	methylated	P2_1_2_1_2_1_	Inward open	None	3.5	0.315	2016-12-14	X-ray	Esser *et al*., 2017
5KOY	M. musculus	mdr1a	ΔLnk34	P2_1_2_1_2_1_	Inward open	ATP to NBD1	3.85	0.289	2016-12-14	X-ray	Esser *et al*., 2017
5KPI	M. musculus	mdr1a		P2_1_2_1_2_1_	Inward open	None	4.01	0.303	2016-12-14	X-ray	Esser *et al*., 2017
5K02	M. musculus	mdr1a	ΔLnk34, E552/1197Q	P2_1_2_1_2_1_	Inward open	Hg ion	3.3	0.285	2016-12-14	X-ray	Esser *et al*., 2017
4XWK	M. musculus	mdr1a	N83/87/90Q, methylated	P2_1_2_1_2_1_	Inward open	PBDE-100	3.5	0.282	2016-04-27	X-ray	Nicklisch *et al*., 2016
4Q9H	M. musculus	mdr1a	methylated	P2_1_2_1_2_1_	Inward open	None	3.40	0.291	2015-03-04	X-ray	Szewczyk *et al*., 2015
4Q9I	M. musculus	mdr1a	methylated	P2_1_2_1_2_1_	Inward open	QZ-Ala	3.78	0.295	2015-03-04	X-ray	Szewczyk *et al*., 2015
4Q9J	M. musculus	mdr1a	methylated	P2_1_2_1_2_1_	Inward open	QZ-Val	3.60	0.282	2015-03-04	X-ray	Szewczyk *et al*., 2015
4Q9K	M. musculus	mdr1a	methylated	P2_1_2_1_2_1_	Inward open	QZ-Leu	3.80	0.321	2015-03-04	X-ray	Szewczyk *et al*., 2015
4Q9L	M. musculus	mdr1a	methylated	P2_1_2_1_2_1_	Inward open	QZ-Phe	3.80	0.293	2015-03-04	X-ray	Szewczyk *et al*., 2015
4M1M	M. musculus	mdr1a		P2_1_2_1_2_1_	Inward open	Hg ion	3.80	0.267	2013-11-13	X-ray	Li *et al*., 2014
4M2S	M. musculus	mdr1a		P2_1_2_1_2_1_	Inward open	QZ-RRR	4.40	0.294	2013-11-13	X-ray	Li *et al*., 2014
4M2T	M. musculus	mdr1a		P2_1_2_1_2_1_	Inward open	QZ-SSS (QZ-Val)	4.40	0.294	2013-11-13	X-ray	Li *et al*., 2014
4KSB	M. musculus	mdr1a		P2_1_2_1_2_1_	Inward open	None	3.80	0.357	2013-07-31	X-ray	Ward *et al*., 2013
4KSC	M. musculus	mdr1a		P2_1_2_1_2_1_	Inward open	None	4.00	0.338	2013-07-31	X-ray	Ward *et al*., 2013
4KSD	M. musculus	mdr1a		P2_1_2_1_2_1_	Inward open	Nanobody Nb592	4.10	0.344	2013-07-31	X-ray	Ward *et al*., 2013
4LSG	M. musculus	mdr1a		P2_1_2_1_2_1_	Inward open	Hg ion	3.80	0.357	2013-07-31	X-ray	c
4F4C	C. elegans	pgp		P2_1_2_1_2_1_	Inward open	None	3.40	0.283	2012-09-26	X-ray	Jin *et al*., 2012
3G5U	M. musculus	mdr1a		P2_1_2_1_2_1_	Inward open	Hg ion	3.80	0.347	2009-03-24	X-ray	Aller *et al*., 2009
3G60	M. musculus	mdr1a		P2_1_2_1_2_1_	Inward open	QZ-RRR	4.40	0.365	2009-03-24	X-ray	Aller *et al*., 2009
3G61	M. musculus	mdr1a		P2_1_2_1_2_1_	Inward open	QZ-SSS (QZ-Val)	4.35	0.356	2009-03-24	X-ray	Aller *et al*., 2009

a – crystallographic space group; b – crystallographic free R factor, a measure of how the model matches diffraction data; c – not published

The definition of P-gp’s four domains was derived from sequence analysis, providing a basic organization of the transporter. The arrival of experimental crystal structures of P-gp brought new structural features to light, which are defined here following the conventions used in the literature. As shown in Figure 1B, the P-gp structure includes two homologous halves: N- and C-terminal halves. For each half, there are two regions: Helix Region and NBD Region. The Helix Region consists of extracellular domains (ECD1 and ECD2), the transmembrane domains (TMD1 and TMD2), and the intracellular domains (ICD1 and ICD2). It should be emphasized that the N-terminal Helix Region contains helices 1, 2, 3, 10, 11 and 6 and that the C-terminal Helix Region includes helices 7, 8, 9, 4, 5 and 12. This structural feature permits the interaction and communication of the two homologous halves at all times.

### Structures of P-gp in different conformations

Although many ABC half transporters have been shown to be functionally similar to P-gp^[67,72,73]^, our discussion of P-gp structures focuses only on those listed in Table 1 due to their very high sequence identity and structural similarity. We believe these proteins have very similar, if not identical, mechanisms of function.

**Figure 2:**
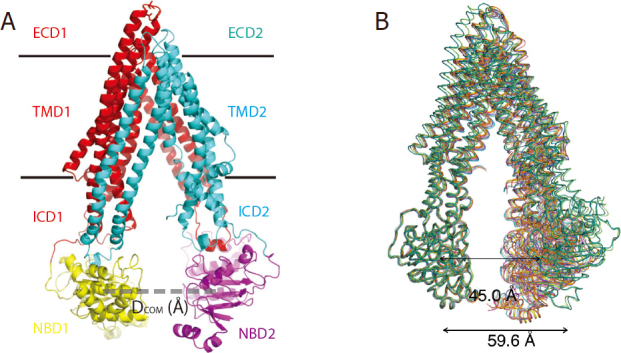
Open-and-close movement of the two halves of P-gp (A) The Definition of D_COM_ is given as the distance (the dotted line) between the center of mass of NBD1 and that of NBD2. (B) Different D_COM_ gap distances among some of the structures of P-gp in the inward-open conformation

The increasing resolution in the published structures of P-gp by different groups employing different methods [Table 1] has significantly improved the quality of P-gp structures over time^[46,64,66,69-71,74]^. In a total of 25 PDB entries [Table 1], one feature is common to all but one^[69]^; they have an inward-open conformation [Figure 1B], meaning that the two NBDs are widely separated and the drug-binding pocket bounded by two TMDs opens towards the cytoplasm. It should be emphasized that not all of the “inward-open” conformations are the same, because the gaps between the two NBDs vary in these structures [Figure 2]. Structures with inward-open conformations have been reported for full-length wild-type *m*P-gp^[46,66,74]^, linker-shortened *m*P-gp^[66]^, methylated *m*P-gp^[65,75]^, chimeric P-gp^[71]^, *m*P-gp bound with various substrates, antibodies and ligands^[65,71,74,76]^, and human and mouse P-gp imbedded in nanodisc^[70]^.

Only one structure of P-gp in the outward-open conformation has been reported, determined at 3.4 Å resolution by cryo-EM^[69]^. This conformation was obtained using a Walker B mutant containing E556/1201Q mutations in the presence of drug vinblastine (150 μM) and Mg•ATP (10 mM). The structure occludes Mg•ATP in both NBDs and does not contain the substrate vinblastine. Thus, it is believed that this represents a post-translocation conformation [Figure 1C]. It should be mentioned that the outward-open conformation structure determined represents a population of only 15% of all the particles selected from the EM micrographs.

### Structures of P-gp complexed with various substrates and inhibitors

The first reported structures of *m*P-gp were not native structures because they contained either bound Hg ions (3G5U) or were in complex with the cyclic peptidyl inhibitors QZ59-RRR (PDB:3G60) or QZ59-SSS (PDB:3G61). QZ59-SSS was later re-named QZ-Val. These structures were re-refined and reported again (PDB:4M1M, 4M2S and 4M2T)^[65]^. In addition to these structures, there is another structure of *m*P-gp, also in an inward-facing conformation and in complex with a nanobody Nb592 (PDB: 4KSD), reported in 2013^[74]^. The nanobody binds to an epitope present at the C-terminal end of NBD1 and strongly inhibits the ATPase activity, presumably by hindering the dimeric association of the NBDs, which is essential for ATP hydrolysis^[74]^. Four co-crystal structures of methylated P-gp with a series of rationally designed cyclopeptides were also presented at lower resolutions (PDB: 4Q9I, RQ9J, 4Q9K and 4Q9L). Interestingly, the binding of some, not all, ligands produces a large conformational change in the TMH4^[75]^.

**Figure 3:**
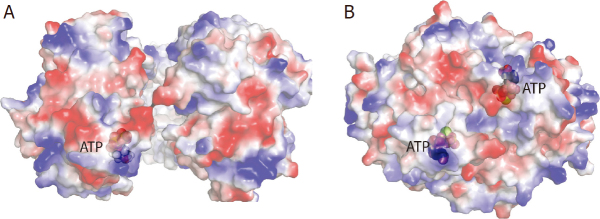
Binding of nucleotides to NBD1 and NBD2 (A) Asymmetric ATP binding to NBD1 in the inward-open conformation obtained from the crystal structure of *m*P-gp (PDB:5KOY). The NBDs are represented by an electrostatic potential surface with positive potential shown in blue, negative potential in red. The bound ATP at the nucleotide-binding site of NBD1 is rendered as a ball-and-stick model and is labeled. (B) ATP binding to both NBDs obtained in the structure with open-outward conformation from a cryo-EM study of *h*P-gp (PDB:6C0V)

More recently, higher resolution structures of *m*P-gp with bound ligands were reported. The improved resolution was achieved by introducing various mutations, chemical modifications or both. One *m*P-gp structure bound with the environmental contaminant polybrominated diphenyl ether (PBDE-100) was determined to 3.5 Å resolution using a triple-mutation (N83/97/90Q) mutant of *m*P-gp that was also methylated^[76]^. Esser and colleagues reported the structure of *m*P-gp with ATP bound asymmetrically at the NBD1; this structure used *m*P-gp with a linker shortened by 34 residues^[66]^ [Figure 3A]. Using a *m*P-gp that has all its extracellular residues replaced by corresponding residues from *h*P-gp and simultaneously stabilized by an engineered disulfide bridge in the NBDs (S559/1204C), Alam *et al*.^[71]^ reported two chimeric P-gp structures determined by the cryo-EM method: one at 4.14 Å resolution with a bound Fab fragment from the *h*P-gp specific monoclonal antibody UIC2 (PDB:6FN4) and another at 3.5 Å resolution bound with the P-gp inhibitor zosuquidar^[71]^. This latter structure was also obtained with the protein embedded in nanodisc, showing zosuquidar in the binding pocket with the same configuration as that in detergent^[70]^. A structure of *h*P-gp embedded in nanodisc and bound with the anticancer drug taxol was also reported, determined by cryo-EM, showing that the drug occupies the same general area for the inhibitor zosuquidar^[70]^.

### Structural conservation of the interface between the NBD and TMD

Although all structures except one determined to date feature an inward-open conformation, the degree of opening or the separation of the two halves of P-gp for these structures is different. One way to describe the degree of opening is to measure the gap distance between the two NBDs. The use of the distance, D_COM_ (Å), between two COMs (center of mass) of NBDs seems most appropriate because it avoids the issue of various orientations of NBDs in different structures [Figure 2A], which could lead to insensitivity to the distances measured by fluorescence or luminescence energy transfer methods for different P-gp conformations [Table 2]. The variations in D_COM_ values obtained for different inward-open structures are illustrated in Figure 2B.

**Table 2:** Gap distances between NBDs measured based on available structures using different methods

PDB	Nucleotide bound	Construct/Substrate/Inhibitor and Other treatment	Distance (Å)	
			COM (NBD1 to NBD2)^a^	CA (N607-T1252)^b^
6C0V	ATP	E/Q	20.8	41.3
6FN1	Apo	Crossed linked/Fab/Zusoquidar	34.6	36.3
5KPI	Apo	WT	45.6	41.7
5KPJ	Apo	WT/ methylated	60.5	66.0

a – COM is center of mass. The distance is measured between the COM of NBD1 to that of NBD2

b – CA is Cα atom of an amino acid residue. The distance is measured between Cα of N607 and T1252 or their equivalent residues in human P-gp.

**Figure 4:**
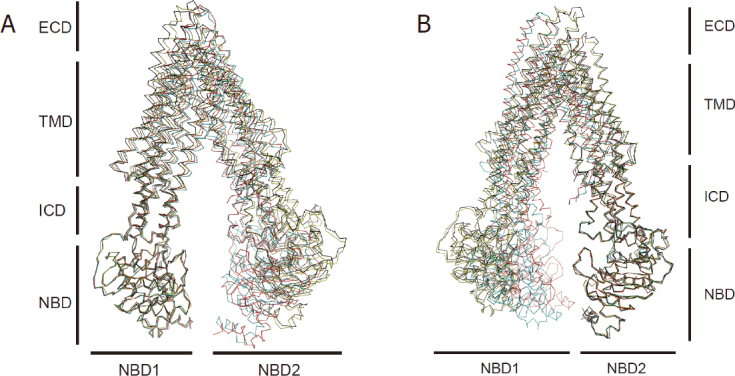
Conserved coupling interface between ICD and NBD (A) Structural alignment of NBD1s from the structures of *m*P-gp with different D_COM_ values. The alignment of NBD1 also brings ICD1 to superposition, whereas the rest of P-gp is not aligned. (B) Here, structural alignment is carried out for NBD2s from structures of *m*P-gp with different D_COM_ values. The alignment of NBD2 also brings ICD2 to superposition, but not the rest of P-gp

When the NBD1 domains of P-gp from structures with different D_COM_ values are superposed, the domains align with each other very well. While this was expected, the ICD1 domains (which were not included in the superposition sets) were also in good agreement, suggesting that the interactions between NBD1 and ICD1 are also maintained in different inward-open conformations [Figure 4A]. By contrast, the TMD1s did not superpose well, and neither did the entire C-terminal half of the molecule (TMD2 and NBD2). In particular, the rms deviations for the structure superposition of TMD1s are proportional to the gaps between the two NBDs. In other words, the larger the gap (D_COM_) is between the two NBDs, the greater the rms deviation is in the alignment of the TMD1s. This conserved interaction between NBD1 and ICD1 is also observed for the C-terminal half of P-gp when the NBD2s are aligned^[66]^ [Figure 4B]. Significantly, the conservation of the ICD/NBD interfaces is largely unchanged when the protein switches from the inward-open conformation to the outward-open conformation^[69]^. Conceivably, the NBD/ICD interfaces of P-gp are important in transmitting conformational changes associated with ATP binding/hydrolysis to substrate translocation and vice versa.

This conserved ICD/NBD interface appears to be unique to P-gp because bacterial ABC transporters feature a more flexible ICD/NBD interface. For example, alignment of multiple structures of the maltose importer showed that the coupling helix rotates relative to the NBDs during a transport cycle^[76,77]^, which resembles a ball-and-socket joint.

### Structural basis of P-gp function

#### Physiological role of the opening and closing movement of the two P-gp halves

The 75-residue linker connecting the two halves of P-gp was originally defined based on sequence alignment of the two halves^[78]^. Analysis of available crystal structures supports the length of the linker to be around 60 residues based on the disordered residues in *m*P-gp structures between the N- and C-terminal halves [Figure 1A]. The function of the linker has been probed genetically and biochemically. For example, when the linker was proteolytically cut, the protein displayed an altered substrate specificity and showed higher basal ATPase activity^[79]^. Genetically shortening the linker by 34 residues led to the loss of P-gp function but the protein appeared to be normally expressed on the cell surface. Replacement of this 34-residue fragment with a helix also inactivated the protein but substituting it with a flexible sequence preserved the P-gp function, suggesting that the flexibility of the linker is needed for its function^[78]^. This requirement of linker flexibility is in agreement with the observation that the linker is essentially not visible in any of the available structures. Moreover, the flexibility of the linker was deemed to be a contributing factor for the low-resolution of full-length *m*P-gp structures^[66]^.

In an attempt to obtain higher resolution structures in an outward-open conformation, Esser and colleagues created a *m*P-gp mutant (^lnk^*m*P-gp) with a linker shortened by 34 residues. The purified ^lnk^*m*P-gp displayed slightly elevated basal ATPase activity but lost drug-stimulated ATPase activity and showed no drug transport *in vivo*^[66]^. Surprisingly, the structure of the ^lnk^*m*P-gp still has the inward-open conformation and is structurally identical to wild-type *m*P-gp reported earlier. Thus, it was concluded that the loss of the drug-stimulated ATPase and transport activity of ^lnk^*m*P-gp was not due to structural damage to the protein but rather to the loss of flexibility, as it was unable to undergo the open-and-close motion. Furthermore, this experiment suggests not only that the opening-and-closing motion of P-gp’s two halves is important for transport function, but that the two halves need to open even wider than we saw in the wild-type structure in order to function. This result also demonstrates that unlike bacterial ABC transporters, in which the two NBDs are often seen staying close together^[80,81]^, P-gp functions very differently and requires a relatively large separation of the two NBDs. Indeed, the gap between the two NBDs measured by the double electron-electron resonance technique in solution for apo-*m*Pgp was consistently larger than that of crystal structures^[82]^.

#### Symmetric vs. asymmetric nucleotide binding

In crystals of ^lnk^*m*P-gp, in the presence of ATP (18 mM), the P-gp structure revealed a molecule of ATP bound asymmetrically to NBD1 but not to NBD2 [Figure 3A]. Other than the shortened linker, ^lnk^*m*P-gp has intact NBDs. This asymmetric ATP binding does not appear to be the result of crystal lattice contacts because there are two independent P-gp molecules in a crystal asymmetric unit and both have ATP occupying only NBD1^[66]^. Despite the difference in ATP binding and in the local environment around the bound nucleotide, the overall structures of the two NBDs are very similar. This structure, in the absence of bound substrate, not only confirmed the one-to-one stoichiometry ratio of nucleotide binding to P-gp, consistent with prior biochemical observations^[36,61,63,66,83-86]^, but also established the order of the reaction sequence for basal ATPase activity, in which the NBD1 is the site that first accepts a nucleotide.

The asymmetric nucleotide binding was supported by the protection of the Walker A lysine residue (K429) in NBD1 from reductive methylation in wild-type *m*P-gp in the presence of nucleotide and by the asymmetric modification of K1072 in NBD2^[66]^. When wild-type *m*P-gp was treated for methylation of lysine residues in the presence of AMP-PNP and subsequently crystallized, the structure of the methylated *m*P-gp displayed a very large separation distance (D_COM_ ~60 Å) of its two NBDs. Perhaps most interestingly, the difference Fourier map showed a density fragment in the nucleotide-binding site of NBD2, not NBD1, that can be interpreted as a tri-methylated Walker A lysine (K1072). This result suggests that during the methylation of P-gp in the presence of AMP-PNP, the NBD1 site was occupied by the nucleotide and thus its Walker A lysine residue K429 was protected from being modified, demonstrating that for wild-type P-gp in solution ATP preferentially binds to the NBD1 in the absence of transporting drugs. In a recently reported experiment using H/D exchange-coupled mass spectrometry of P-gp solubilized in detergent or reconstituted into nanodics, it was only NBD2, not NBD1, that consistently showed increased H/D exchange when the protein was in the vanadate-trapped state, which is in agreement with asymmetric function of NBDs^[87]^.

The *h*P-gp structure in outward-open conformation has both NBDs bound with ATP^[69]^, bringing the two NBDs together to form a closed dimer and re-orienting the drug-binding cavity observed in the inward-open structures toward the extracellular space [Figure 3B]. The discrepancy between the EM structure and available biochemical data with respect to the asymmetric nucleotide binding was attributed to different experimental conditions. While the cryo-EM grids were prepared with saturating amounts of ATP (10 mM), in the biochemical studies, the stoichiometry values were determined after affinity chromatography in an ATP-free buffer^[88]^. It is also worth noting that the outward-open conformation EM structure was reconstructed from only a fraction (15%) of all P-gp particles selected from cryo-EM 3D classification analysis. Thus, in wild-type P-gp, the outward-open state requires the binding of nucleotide to both sites. This state apparently is very short-lived^[88]^ and is called the transition state. However, this transient state is significantly stabilized in the double Walker B mutant (human E556/1201Q; mouse E552/1197Q)^[56]^.

#### The open-and-close motion of the two halves of P-gp is correlated with the movement of each individual helix in the TMD

P-gp is a very flexible molecule; the flexibility is manifest in structures of *m*P-gp with respect to the opening-and-closing of the two halves, as obtained by cryo-EM, from different crystal forms and even in molecules from the same crystals^[66,71,76]^, in cross-linking experiments^[89]^, and in various fluorescent^[54,90]^ and EPR measurements^[82,90]^. This open-and-close movement can be quantitatively measured using the D_COM_ (Å) metric, the distance between the two COMs (center of mass) of the NBDs in different structures [Figure 2A]. The representative D_COM_ values obtained for different structures are illustrated in Figure 2B.

**Figure 5:**
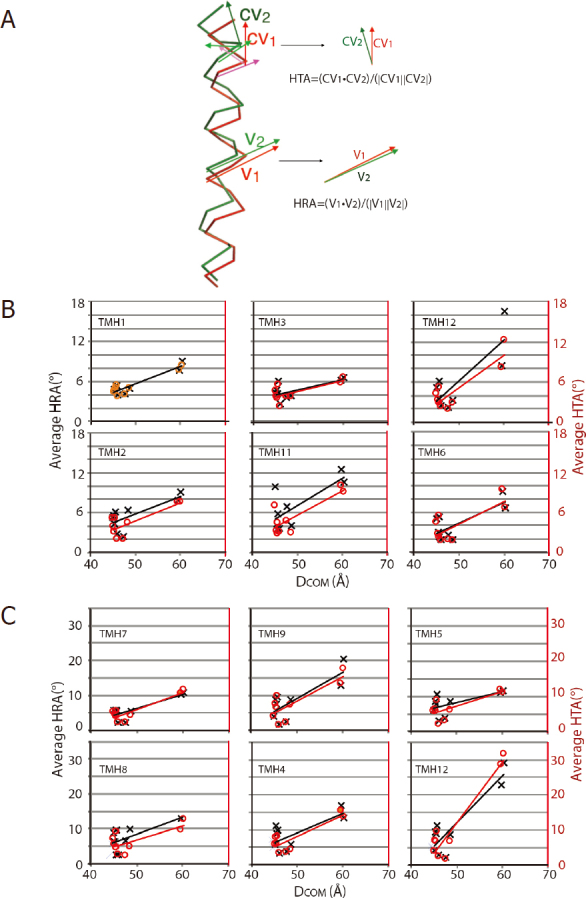
Coupled conformational changes in TMDs (A) Definition of the average helix rotation angle <HRA> and the average helix tilting angle <HTA>. After superposition of either NBD1 or NBD2, pairs of aligned helices are analyzed with one serving as a reference helix. The helix rotation angle (HRA, in degree) is defined as the angle between a pair of vectors from two equivalent, aligned helices (red and green). Each vector v1 (red) or v2 (green) joins the two neighboring CA atoms. The average helix rotation angle <HRA> is the average over all HRAs defining the length of the helix. The helix tilt angle (HTA, in degree) is computed between a pair of cross-vectors cv1 and cv2 from the two equivalent helices. Each cross-vector is defined by three consecutive CA atoms of a helix. The average helix tilt angle <HTA> is the average of all HTAs over the length of the helix. (B) correlation of <HRA> and <HTA> with DCOM for helices in the TMD1 domain (C) correlation of <HRA> and <HTA> with DCOM for helices in the TMD2 domain

By superposing pair-wise structures of *m*P-gp with different D_COM_ values, Esser and colleagues observed that the D_COM_ values and rms deviations of the TMD alignments are correlated^[66]^, which means that the greater the difference in D_COM_s is between the two P-gp structures, the larger the conformational changes are in the TMDs of the two structures. Thus, it is interesting to know whether the open-and-close motion of P-gp brings changes to the drug binding site. We now know it does. TMD conformational change consists of structural changes that are beyond the conventional definition of the movement of individual, rigid domains^[66]^. The conformational changes of P-gp TMDs involves in, in addition to whole domain movement, relative movements of individual helices with respect to each other within the domain. The movements of individual helices of one P-gp structure (with one D_COM_ value) are complex compared to those in another structure of a different conformation (with another D_COM_ value) and in part can be described quantitatively as average helix rotation angle (<HRA>) and average helix tilting angle (<HTA>) [Figure 5A] that are defined with respect to corresponding helices of a reference structure (inward-open conformation, PDB: 6C0V). From the quantitative analysis of the helix movement^[66]^, the following conclusions can be reached: (1) individual helices in both TMD1 and TMD2 rotate and tilt as the two halves of P-gp undergoing the open-and-close movements; (2) Helices in TMD2 rotate and tilt more than those in TMD1; (3) In the N-terminal half, TMH10 and TMH11 have the most rotation and tilting; (4) In the C-terminal half, TMH4 and TMH12 show the most movement; (5) Most significantly, the D_COM_ values is highly correlated to the <HRA> and <HTA> [Figure 5B].

#### Mechanism of polyspecific drug recognition: The Dynamic Conformation Sampling Hypothesis

It is well known that P-gp can transport numerous structurally and chemically distinct xenobiotics such as cancer drugs. As an example, the Canadian drugbank lists 231 drugs currently in clinical use as P-gp substrates (https://www.drugbank.ca/categories/DBCAT002668). P-gp has also been shown to bind multiple drug molecules simultaneously^[91]^. The mechanistic basis for this broad specificity and binding multiplicity of P-gp remains elusive; the main issue is the underlying chemistry involving substrate recognition by P-gp, which has to be specific to prevent free movement of materials in and out of cells. From the analysis given in the preceding sections, a novel mechanism for the polyspecificity of P-gp substrate recognition can be proposed.

Since the ^lnk^*m*P-gp is structurally intact, remains in an inward-open conformation, possesses basal ATPase activity, and yet is unable to transport substrate, it is reasonable to suggest that the ^lnk^*m*P-gp is conformationally restricted, and cannot open the two halves to the fullest extent. Thus, the open-and-close movement must be an intrinsic property of P-gp function that takes place spontaneously even in the absence of substrate. Indeed, many experiments in the literature support the open-and-close movement of P-gp: (1) H/D exchange-coupled mass spectrometry demonstrates that the so-called EX1 or bimodal kinetics occurs on different time scales from seconds to hours, suggesting a highly complex but correlated motion of the two halves of P-gp^[87]^; (2) cryo-EM images of P-gp in the presence or absence of ATP show a wide range of separations between the two halves of the molecules^[69,92,93]^; (3) Time-resolved atomic force microscopy correlates the up-and-down motion of P-gp embedded in the membrane bilayer to the open-and-close movement of the two halves; this up-and-down motion also takes place in the absence of ATP^[94]^; and (4) EPR double electron-electron resonance also demonstrated large separations of the two NBDs during the catalytic cycle of P-gp^[82]^.

The strong correlation observed between the open-and-close movement of the two lobes of P-gp and each individual helix rotation and tilting suggests that P-gp’s drug-binding pocket is quite fluid, changing in shape, size and surface properties as the two halves undergo the open-and-close movement. It was hypothesized that under resting conditions, P-gp embedded in the membrane bilayer undergoes a slow open-and-close or breathing motion of its two lobes. The rate of this motion is no less than the basal ATPase activity, which has a turnover rate of 3-4 ATPs per minute. As the two lobes of P-gp move in and out, helices in the TMDs rotate, tilt and slide up and down with respect to each other, presumably in a programed fashion. These coordinated movements result in a continuously changing substrate-binding pocket landscape, which could facilitate interaction with molecules that have diverse structures and can theoretically be large in number. However, such as a system may inevitably be an inefficient transporter with respect to binding affinity. This model for the polyspecificity of P-gp was termed the “Dynamic Conformation Sampling Hypothesis”^[66]^. Concomitantly, this hypothesis assigns a functional necessity to the open-and-close motion of the two lobes of the molecule and provides an explanation for the origin of basal ATPase activity.

The Dynamic Conformation Sampling Hypothesis shares some features with the “Induced Fit Mechanism”^[41]^, which postulates that a substrate creates its own binding environment, requiring molecular flexibility. However, there are fundamental differences between the two. In the Dynamic Conformation Sampling Model, the binding surface is not induced by a substrate but is rather created to scout various conformations as P-gp undergoes the open-and-close movement. This model also requires the protein to undergo constant and spontaneous open-and-close movement that drives conformational changes in the drug-binding pocket.

It is generally agreed that the mechanism of substrate transport by P-gp falls into the general scheme of an alternating access model^[60]^. Accordingly, the P-gp molecule ought to have an inward-open and an outward-open conformation. Structural studies of bacterial transporters such as MsbA, Sav1866, McjD and MJ1267 showed a strong tendency for transporters to take an outward-open conformation in the presence of nucleotide^[67,73,95,96]^. Thus, the idea that a P-gp molecule spends significant portion of its time in an outward-open conformation and then occasionally opens the two NBDs for the purpose of substrate uptake has been suggested, because it seems in accordance with the fact that there is 2-4 mM ATP in the cytosol and appears corroborated by several biochemical and molecular dynamics studies^[53,97]^. This notion of P-gp being mostly in the outward-open conformation is contradicted by all but one high-resolution structure and by low-resolution EM studies that show P-gp, on an EM grid, mostly in the inward-open conformation^[92,93]^. Even in the presence of a drug substrate and a high concentration of Mg•ATP, P-gp in the outward-open conformation represents only 15% of the total population, as revealed by recent high-resolution cryo-EM study of human P-gp^[69]^.

#### Support for the Dynamic Conformation Sampling Hypothesis

**Figure 6:**
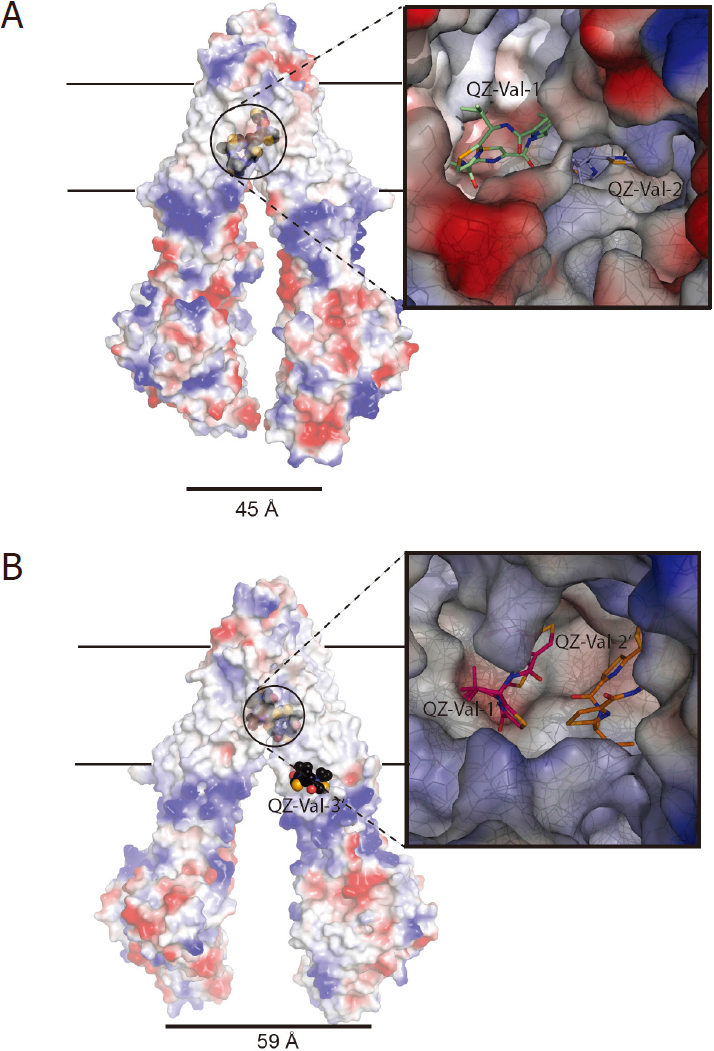
Plasticity of P-gp’s drug-binding surface (A) The binding environment of the cyclic inhibitor QZ-Val in the *m*P-gp structure with D_COM_ of 45 Å (PDB:4M2T). The structure is rendered as a semi-transparent electrostatic surface with bound QZ-Val shown as stick models. The membrane bilayer is indicated by the two parallel horizontal lines. The inset is a magnified inhibitor binding environment viewed from the cytosolic side of P-gp toward the extracellular side. The bound whole QZ-Val is labeled as QZ-Val-1 and the half molecule is labeled as QZ-Val-2. (B) A different binding environment of the cyclic inhibitor QZ-Val in the *m*P-gp structure with D_COM_ of 60 Å (PDB:4Q9J). There are three modeled QZ-Val molecules labeled as QZ-Val-1’, QZ-Val-2’ and QZ-Val-3’

The Dynamic Conformation Sampling Hypothesis predicts the following properties for P-gp, which can be verified by experimental approaches: (1) basal ATPase activity is an intrinsic property of P-gp function reflecting the minimum rate of the open-and-close movement of the two lobes of P-gp. It should be inversely proportional to the length of the linker, which is consistent with the observation that ^lnk^*m*P-gp exhibits elevated basal ATPase activity^[66]^; (2) the drug binding surface of P-gp is fluid, suggesting that there is no fixed drug-binding pocket. For a given drug there may exist more than one binding site and it may bind to the protein with different conformations. The existing structures of P-gp in complex with various compounds support this notion. One example is the binding of the cyclic peptide QZ-Val, which occurs in at least two different P-gp conformations: one with a D_COM_ of 45 Å (PDB:4M2T) and another for methylated *m*P-gp with a D_COM_ of 60 Å (PDB:4Q9J)^[65,75]^. For the PDB:4M2T, there are one and a half molecules of QZ-Val bound in the putative drug-binding pocket, each with a very different binding environment [Figure 6A]. The structure of methylated P-gp has three bound QZ-Val, two binding in the substrate biding pocket [Figure 6B] and the other binding outside the pocket. It should be pointed out that by photolabeling experiments, photoactive substrates such as Azodopin or IAAP were able to label P-gp in many different TM locations, which is consistent with the Dynamic Conformation Sampling Hypothesis^[43,44,48]^. Binding of multiple drugs has also been suggested by cross-linking experiments^[91]^; (3) relative rotations of individual TM helices were also inferenced for P-gp by cross-linking experiments. Using a set of Cys mutations in TM12 (V982C) and TM6 (L339C) (V978C/L335C in mouse) in a Cys-less P-gp background, Loo and colleagues observed cross-linking of the protein only in the absence of ATP but not in the presence of ATP. Using a slightly different mutation in TM6 F343C and the same mutation in TM12 (V982C), the authors observed that the protein was able to cross-link in the presence ATP. Thus, a helix rotation of either helix or both must be involved^[98]^. In a recent experiment involving H/D exchange-coupled mass spectrometry, it was demonstrated that the rates of H/D exchange for different TM helices vary depending upon the conformation of P-gp, suggesting movement of TM helices during catalytic cycles^[87]^.

### Future perspective

The interest in elucidating the mechanism of P-gp function is rooted deeply in our desire to understand how the expression of P-gp affects drug efficacies and in our effort to circumvent its effect under clinical conditions. To achieve our goals, precise knowledge regarding the ways P-gp is activated and inhibited is critical, requiring structural information at atomic resolution. Decades of efforts to determine the structure of P-gp by many laboratories has already yielded rich information, contributing to our understanding of the structure and function relationship of P-gp. The recent development of the cryo-EM method in the field of structure biology will surely accelerate this process.

Despite the progress made, issues remain in the study of P-gp function: (1) there is no full understanding at the cellular level of how P-gp expression is regulated and effort is needed to elucidate the signaling cascades controlling its expression upon cellular exposure to toxic environments; (2) to study the mechanism of P-gp function, there is a need to establish a comprehensive model describing its function, from substrate entry to efflux. Such a model should be able to integrate available published research and provide strategies for the control of P-gp activity. Towards that goal, researchers need to work out the details at high resolution of every step of P-gp function, including (i) Substrate recognition, selection and the origin of binding affinity, (ii) The mechanism for substrate-stimulated ATPase activity, including how the substrate binding signal is transmitted to the NBDs and the sequence of events that follows leading to ATP hydrolysis. (iii) Understanding at atomic resolution P-gp’s characteristic activation/inhibition bell-shaped ATPase profile. (iv) Substrate release; (3) little is known about regulation of P-gp activity at the protein level, and whether there are cellular factors capable of controlling the function of P-gp; (4) Ultimately, there is a need to control the function of P-gp by synthetic inhibitors that can be obtained by rational design afforded by the available high-resolution P-gp structures or by experimental screening.

## Declarations

### Availability of data and materials

The data were strictly obtained from medical records according to the privacy policy and ethics code of our institute.

### Copyright

© The Author(s) 2019.
